# Projected Return on Investment From Implementation of a Lifestyle Intervention to Reduce Adverse Pregnancy Outcomes

**DOI:** 10.1001/jamanetworkopen.2022.30683

**Published:** 2022-09-06

**Authors:** Melanie Lloyd, Helena Teede, Cate Bailey, Emily Callander, Zanfina Ademi

**Affiliations:** 1 Centre for Medicine Use and Safety, Faculty of Pharmacy and Pharmaceutical Sciences, Monash University, Melbourne, Victoria, Australia; 2 School of Public Health and Preventative Medicine, Monash University, Melbourne, Victoria, Australia; 3 Centre for Health Research and Implementation, Monash University, Melbourne, Victoria, Australia; 4 Health Economics Unit, Melbourne School of Population and Global Health, University of Melbourne, Melbourne, Victoria, Australia

## Abstract

**Importance:**

Structured antenatal diet and physical activity interventions have been shown to be associated with reduced adverse pregnancy outcomes and recommended to be routinely offered to all pregnant women. The health cost implications of population-level implementation are unclear.

**Objective:**

To estimate the budget impact associated with integrating structured diet and physical activity interventions into routine antenatal care.

**Design, Setting, and Participants:**

This economic evaluation was conducted from the perspective of Australian health funders. An open-source decision-tree model was constructed to compare the projected budget outcomes of implementing lifestyle intervention vs usual care. Scenario, deterministic, and probabilistic sensitivity analysis were completed. The study setting was Australian health services, and the study population was all Australian women projected to give birth in the years 2022 to 2026 (approximately 330 000 per year).

**Interventions:**

Structured diet and physical activity intervention provided by trained health professionals, integrated into routine antenatal care. Comparator was usual care, which currently in Australia does not include routine structured lifestyle interventions.

**Main Outcomes and Measures:**

Return on investment (ROI) ratio for lifestyle intervention (cost of intervention divided by cost savings attributable to reduced maternal and infant adverse events) from the perspective of Australian health care funders. Adverse events were obtained from a published meta-analysis and population data. Costs were estimated from aggregate trial data and clinical pathways and valued at the year incurred.

**Results:**

Intervention offered an ROI ratio of 4.75 over the 5-year program; hence every Australian dollar spent on implementation produced an estimated return of A$4.75. The projected total 5-year intervention cost was A$205 million ($151 million), with cost offsets (from reduced incidence of adverse pregnancy outcomes) of A$1022 million ($755 million), and health budget savings of A$807 million (95% CI, A$129 million to A$1639 million) ($596 million [95% CI, $95 million to $1211 million]); 93.3% of the 10 000 iterations were within cost saving, and results were robust to scenario and sensitivity analyses.

**Conclusions and Relevance:**

This economic evaluation found that providing access to structured diet and physical activity lifestyle interventions for all pregnant Australian women was estimated to provide strong return on investment for health funders. The open-source model developed can be used by other jurisdictions and health services to explore cost implications of implementation within their patient population.

## Key Points

**Question:**

What are the cost implications for health funders if clinically effective structured antenatal diet and physical activity interventions are routinely implemented?

**Findings:**

This economic evaluation of routine implementation at the Australian population level estimated that A$4.75 would be returned for every 1 Australian dollar invested by health funders, with cost offsets from reduced perinatal morbidity and adverse events exceeding the cost of the intervention.

**Meaning:**

These findings suggest that providing access to structured diet and physical activity lifestyle interventions for all pregnant Australian women may be associated with substantial health and economic benefits.

## Teaser

This economic evaluation assesses the budget impact associated with integrating a structured diet and physical activity intervention into routine antenatal care in Australia.

## Introduction

Globally, around half of pregnancies are affected by excess gestational weight gain, which is strongly and independently associated with adverse maternal and infant outcomes.^1,2^ These outcomes include development of gestational diabetes and hypertensive disease in pregnancy (HDP), together with increased risk of cesarean delivery and/or neonatal intensive care.^1,3,4,5^ In 2019, 48% of pregnant women in Australia were classified as overweight or obese, 11% were diagnosed with gestational diabetes, and 3% with HDP.^6^ Health service costs associated with pregnancy and birth in Australia were 25% higher for women with HDP, and 13% for gestational diabetes, compared with those without these conditions.^7,8^

Antenatal lifestyle interventions (to promote healthy lifestyle choices during pregnancy) have been evaluated in recent systematic reviews, and those including structured diet and physical activity coaching separately or together are shown to be associated with reduced excess gestational weight gain, and limiting associated complications, including gestational diabetes, HDP, cesarean birth, and neonatal intensive care unit (NICU) admission.^4,9^ There was consistent evidence of benefit across maternal body mass index (BMI) categories, and authors of both reviews have recommended that structured lifestyle interventions should be implemented into routine care with the goal of preventing excess gestational weight gain.^4,9^ This recommendation aligns with that of the 2021 US Preventative Services Taskforce.^10^ These interventions have also been shown to be cost-effective, and potentially even cost saving from the perspective of the health service when infant costs such as NICU are considered.^11,12^

Given current constraints on budget, and growing pressures on hospitals to curtail spending, assessment of projected costs at the system level is essential for the planning and management of health budgets. A budget impact analysis (BIA) estimates changes in expenditure within a health system following introduction of a new intervention or technology.^13^ This can inform decision makers by guiding prioritization and driving implementation of cost-effective novel treatments.^14^

This economic evaluation aims to estimate the total impact on Australia’s health budget after integrating routine pregnancy lifestyle interventions (with structured diet and physical activity components) into antenatal care. The cost of the program over a 5-year implementation timeframe is considered from the perspective of health service funders. While the Australian example is used for illustrative purposes, the open source model can be used by any health service or jurisdiction to explore data from their own patient population and setting.

## Methods

### Design

This economic evaluation was undertaken using a decision-tree model comparing structured diet and physical activity interventions with usual care to determine the cost of preventing adverse pregnancy outcomes (eFigure in the Supplement). Pregnant women were assigned to 1 of 4 health states: (1) develop gestational diabetes, (2) develop HDP, (3) develop both gestational diabetes and HDP, (4) develop neither gestational diabetes nor HDP. For each health state, probability of 4 different modes of birth were modeled (cesarean delivery [CD] [planned or after spontaneous onset of labor], induction of labor with vaginal birth [IVB], induction of labor with cesarean [ICD], spontaneous vaginal birth [VB]); together with associated need for NICU or Special Care Nursery (SCN) admission at birth. Costs associated with (1) provision of the lifestyle intervention, (2) antenatal treatment of gestational diabetes and HDP, and (3) birth, were applied at the population level and extrapolated over 5 years (2022-2026) to estimate the budget impact of a nationwide rollout of the intervention. Intervention scale-up over the 5 consecutive years of the program was assumed (allowing for failed uptake in a small number of health services) (Table 1). The budget impact analysis was conducted and reported consistent with published guidelines, without discounting applied.^13^ Results are presented in Australian dollars (A$), converted to US dollars ($) using the International Monetary Fund exchange rate for 22 March 2022 of 0.739.^15^ The study is reported consistent with the Consolidated Health Economic Evaluation Reporting Standards (CHEERS) reporting guideline. Use of Maternity1000 data use was approved by The Townsville Hospital and Health Service Human Research Ethics Committee and the Australian Institute of Health and Welfare Ethics Committee.

**Table 1.** Base Case Input Parameters, Parameter Variation, and Their Distribution

Year	No. of pregnancies in Australia	%		Total women receiving intervention
		Proportion of caseload included in rollout	Proportion of eligible women attending	
2022	327 222	30	80	78 533
2023	328 805	50	80	131 522
2024	329 749	70	80	184 659
2025	330 089	90	80	237 664
2026	329 850	90	80	237 492
**Projected 2022 prices, A$ ($)^a^**				
**Health Cost**	**Mean**	**−30%**	**+30%**	**Distribution**
Antenatal costs				
GD	1113 (823)	779 (576)	1447 (1069)	Gamma
HDP	2029 (1499)	1421 (1050)	2638 (1949)	Gamma
GD and HDP	2935 (2169)	2055 (1519)	3816 (2820)	Gamma
Vaginal birth cost	7261 (5366)	5083 (3756)	9439 (6975)	Gamma
Cesarean cost	13 361 (9874)	9352 (6911)	17 369 (12 836)	Gamma
Induction with vaginal birth cost	9984 (7378)	6989 (5165)	12 979 (9591)	Gamma
Induction with cesarean cost	16 084 (11 886)	11 259 (8320)	20 909 (15 451)	Gamma
NICU/SCN admission cost	24 073 (17 790)	16 851 (12 453)	31 295 (23 127)	Gamma
**Intervention effect sizes (diet and/or physical activity intervention)**				
**Ratio**	**Mean (95% CI)**			**Distribution**
GD risk ratio	0.670 (0.58-0.77)			Log normal
HDP risk ratio	0.737 (0.60-0.89)			Log normal
GD and HDP risk ratio	0.670 (0.58-0.77)			Log normal
Cesarean risk ratio	0.929 (0.86-1.01)			Log normal
Induction risk ratio	1			
NICU/SCN risk ratio	0.795 (0.64-0.98)			Log normal

Abbreviations: A$, Australian dollar; GD, gestational diabetes; HDP, hypertensive disease in pregnancy; NICU, neonatal intensive care unit; SCN, special care nursery; $, US dollar.

^a^ Values converted from A$ to $ by multiplying by exchange rate (0.739 as of March 22, 2022).^15^

### Population

The model requires estimates for the number of births per year, as well as the characteristics of these births. For our Australian example, the estimated number of births per year were taken from Australian Bureau of Statistics projections (see Table 1).^16^ Multiple births (1.5% of total pregnancies) were accounted for when estimating the number of pregnancies (and therefore number of women eligible for the intervention).^17^ In 2019, the average maternal age in Australia was 30.8 years, and 47.5% of pregnant women were overweight or obese.^6^

Inputs under usual care were derived from a Monash Health routine maternity care data set.^12^ Monash Health, in Melbourne, is Australia’s largest public health service and manages more than 11 000 births per year from an ethnically diverse population at multiple hospitals. Representative data from all singleton pregnancies resulting in births of more than 20 weeks’ gestation were collected between 2009 and 2013 (approximately 38 000).

### Pregnancy Outcomes Under Current Standard Care

In the Monash Health data set, 46.6% of pregnant women were overweight or obese (BMI >25), 7.5% were diagnosed with gestational diabetes, 3.8% with HDP, and 0.5% with both (Table 2). These percentages are consistent with those observed in the wider Australian population.^6^ Overall, 27.8% of births were via CS, and 21.9% were induced,^12^ and the percentages assigned to each mode of birth, according to antenatal health state, have been published previously.^11^ At the time of data collection, pregnant women received no structured diet or physical activity interventions targeting healthy lifestyle.^12^

**Table 2.** Risk of Event in Control and Intervention Groups, and Percentage of Cases Avoided in Intervention Group

Variable	%		
	Control	Intervention	Cases avoided
Diet and/or PA			
Gestational diabetes	7.50	5.02	2.48
HDP	3.80	2.80	1.00
Both	0.50	0.33	0.17
Cesarean delivery	22.05	20.34	1.71
Induction-vaginal delivery	16.10	15.59	0.51
Induction-cesarean	5.67	5.02	0.65
NICU/SCN admission	18.00	14.30	3.70
Diet only			
Gestational diabetes	7.50	4.92	2.58
HDP	3.80	3.08	0.72
Both	0.50	0.33	0.17
Cesarean delivery	22.05	22.96	−0.91
Induction-vaginal delivery	16.10	15.66	0.44
Induction-cesarean	5.67	5.71	−0.04
NICU/SCN admission	18.00	12.60	5.40
PA only			
Gestational diabetes	7.50	4.68	2.82
HDP	3.80	2.57	1.23
Both	0.50	0.31	0.19
Cesarean delivery	22.05	19.22	2.83
Induction-vaginal delivery	16.10	15.49	0.61
Induction-cesarean	5.67	4.71	0.96
NICU/SCN admission	18.00	13.15	4.85
Diet and PA			
Gestational diabetes	7.50	5.62	1.88
HDP	3.80	2.90	0.90
Both	0.50	0.37	0.13
Cesarean delivery	22.05	21.23	0.82
Induction-vaginal delivery	16.10	15.67	0.43
Induction-cesarean	5.67	5.28	0.39
NICU/SCN admission	18.00	17.21	0.79
Unstructured			
Gestational diabetes	7.50	7.69	−0.19
HDP	3.80	4.27	−0.47
Both	0.50	0.51	−0.01
Cesarean delivery	22.05	21.77	0.28
Induction-vaginal delivery	16.10	16.25	−0.15
Induction-cesarean	5.67	5.67	0.00
NICU admission	18.00	19.67	−1.67

Abbreviations: HDP, hypertensive disease in pregnancy; NICU, neonatal intensive care unit; PA, physical activity; SCN, special care nursery.

Risk of admission to NICU or SCN under usual care (18% of births) was taken from the publicly available Australian Perinatal data collection 2018 to 2019.^6^ Results of the BIA both incorporating and excluding NICU/SCN admission were calculated, given the impact this parameter had on results in previous cost-effectiveness modeling.^12^

### Health Costs

Unit costs are required for mode of birth, induction of labor, antenatal care for gestational diabetes and HDP, and NICU and/or SCN admission. Costs for different modes of birth were obtained from diagnosis-related group (DRG) data from 2018 to 2019.^18^ The weighted cost per DRG provides an indicator of mean cost per separation for vaginal birth and CS (eTable 5 in the Supplement). Costs of antenatal care for gestational diabetes and HDP were derived from patient pathways developed for the earlier cost-effectiveness analysis, incorporating relevant item costs from the Medical Benefits Scheme (MBS), Pharmaceutical Benefits Scheme (PBS), and antenatal hospital admission DRGs (eTables 1-4 in the Supplement).^11^ All costs were inflated to projected 2022 to 2026 prices using the long-term trend (2.0%) in health price index^19^ (Table 1).

Costs included in the pathways were further subcategorized into outpatient (MBS and PBS items) and inpatient (costs of hospital admission and inpatient procedures DRGs) categories, to determine the relative proportion of cost savings attributable to alternative funding pools (eTables 2-6 in the Supplement).

### Intervention Design and Costs

Lifestyle interventions incorporating structured dietary or physical activity components, delivered separately or together, with or without behavioral change, are associated with reduced adverse pregnancy outcomes.^4,9^ Core elements of interventions linked to efficacy (session duration, frequency, intensity and mode of delivery) are being explored through framework analysis, and behavioral change technique taxonomy.^20^ It is recognized that flexible implementation of these interventions across different settings, populations and health systems is desirable, provided core elements such as delivery by additional trained health professionals are retained.

Intervention costing for this analysis incorporates appropriately trained health coaches, employed to deliver the lifestyle intervention within diverse health systems. The intervention could be delivered by trained health professionals including dieticians, exercise physiologists, midwives, or physiotherapists. Full-time annual salary costs of A$82 600 ($61 041) per coach (2022 prices, inflated in subsequent years by 3.35% to allow for wage growth) were applied, based on a midpoint of published midcareer salary bands for dieticians, physiotherapists, and midwives in different Australian states and territories (eTable 9 in the Supplement). Salary on-costs of 15%, and a fixed cost allowance of 20% (for training, facility hire, information technology support, and administrative resources) were also incorporated. An average caseload allocation of 500 women per full-time equivalent coach per year was assigned in the base case, consistent with advice from antenatal service clinicians. In the base case analysis 80% attendance within the eligible population was applied as a conservative measure.^21^ Intervention cost per women was A$228 ($168; 2022 prices).

### Outcomes Associated With Intervention

The association of the intervention on risk of adverse outcome (gestational diabetes, HDP, CS, NICU and/or SCN admission) was obtained from our recently published systematic review and meta-analysis.^9^ In the meta-analysis, interventions applied in underlying RCTs were categorized as: (1) structured diet alone (Diet), (2) structured physical activity alone (PA), (3) structured diet and physical activity (diet and PA), (4) structured diet and/or physical activity (diet and/or PA), or (5) unstructured (comprised of mostly written educational materials, weight monitoring, behavioral strategies alone, or inadequately described). For this BIA, diet and/or PA intervention was adopted as the base case. Odds ratios from the meta-analysis were converted to risk ratios (RR) as per the method documented previously.^9,11^ Mean RRs, 95% CIs, and distributions are presented in Table 1 (eTable 10 in the Supplement). RRs for induction were set at 1 as this information was not included in the meta-analysis.

### Statistical Analysis

The cost of providing the intervention as per the base case assumptions from each year (2022 to 2026) was calculated. The intervention cost was then offset by the change in health costs (antenatal care and delivery) resulting from implementation of the intervention package. Aggregate costs (or cost savings) for each year were derived, together with the cost (saving) per eligible pregnant woman receiving the intervention per year. A return on investment (ROI) ratio was calculated to determine the return (in terms of health cost savings) for each dollar spent providing the intervention.

Analysis of baseline health states and demographic data from the Monash Health and Maternity1000 data sets was completed using STATA version 16.1 (StataCorp); decision tree and budget impact modeling, together with sensitivity analyses, were completed in Microsoft Excel with @RISK Analysis Add-in Industrial Edition 8.1.1 (Microsoft Corp) from September 2021 to February 2022.

#### Scenario Analyses Using Alternative Data Source

To investigate external validity of the model results, analyses were repeated using data from the Maternity1000 Queensland perinatal data set 2012 to 2019 (eTable 7 and 8 in the Supplement). Over this period, structured physical activity and diet counselling interventions were not a component of routine antenatal care in Queensland.^22^ Of women included in the data set, 9.6% had gestational diabetes, 2.9% had HDP, and 0.1% both; 41.9% were overweight or obese, 33.7% had a cesarean delivery, and 18.3% of pregnancies resulted in NICU/SCN admission. In scenario 1, model parameters were updated for health states and birth outcomes under usual care with baseline risks obtained from Maternity1000 (eTable 11 in the Supplement). Scenario 2 also included parameter distributions for health costs derived from individual patient cost data (eTable 7 and 8 in the Supplement).

#### Sensitivity Analysis

One-way sensitivity analysis was performed with results presented in a tornado diagram to demonstrate the extent to which the base case budget impact is sensitive to change in underlying parameters. Two-way sensitivity analysis was conducted to explore the association between intervention cost and the number of women managed by each health coach each year.

Probabilistic sensitivity analyses (10 000 iterations) were conducted for the diet and/or PA intervention grouping to estimate the impact of uncertainty around the cost and effect parameters. Consistent with the underlying cost-effectiveness analysis, risk ratios for association were varied by 95% CIs (log-normal distribution) and costs by plus or minus 10% (gamma distribution).^12^

## Results

### Base Case

The diet and/or PA intervention offered a ROI ratio of 4.75 over the 5-year program, therefore every Australian dollar spent on the program yielded an estimated return of 4.75 Australian dollars (Table 3). This is derived from a total intervention cost of A$205 million ($151 million), cost offsets (from reduced incidence of maternal morbidity and adverse outcomes) of A$1022 million ($755 million), and health budget savings of A$807 million ($596 million). Cost saving per woman was estimated at A$951 ($703) in the final year of the program (2026). The unstructured intervention grouping was the only type to not deliver a ROI ratio greater than 1.

**Table 3.** Total Estimated Budget Impact and Cost Per Woman (With and Without NICU and SCN)^a^

Variable	Cost, A$						ROI ratio^b^
	2022	2023	2024	2025	2026	Total	
Total cost (including NICU and SCN)							
Diet and/or PA	−69 859 651	−118 961 232	−169 820 949	−222 215 601	−225 751 261	−806 608 694	4.75
Diet	−84 314 855	−143 653 978	−205 183 408	−268 638 744	−273 068 664	−974 859 649	5.54
PA	−100 264 772	−170 900 032	−244 202 457	−319 862 186	−325 278 836	−1 160 508 284	6.40
Diet and PA	−7 935 519	−13 180 857	−18 332 967	−23 345 151	−23 049 919	−85 844 412	1.40
Unstructured	49 443 071	84 834 708	122 035 035	160 927 209	164 772 125	582 012 148	−1.71
Cost per woman (including NICU and SCN)							
Diet and/or PA	−890	−904	−920	−935	−951	NA	NA
Diet	−1074	−1092	−1111	−1130	−1150	NA	NA
PA	−1277	−1299	−1322	−1346	−1370	NA	NA
Diet and PA	−101	−100	−99	−98	−97	NA	NA
Unstructured	630	645	661	677	694	NA	NA
Total cost (excluding NICU and SCN)							
Diet and/or PA	11 461	394 376	1 108 112	2 176 716	2 963 582	6 654 246	0.97
Diet	17 787 114	30 759 198	44 593 546	59 263 541	61 150 085	213 553 485	−0.01
PA	−8 483 374	−14 116 717	−19 673 211	−25 104 597	−24 843 258	−92 221 157	1.43
Diet and PA	6 963 682	12 270 337	18 115 665	24 503 898	25 720 858	87 574 439	0.59
Unstructured	17 826 561	30 826 582	44 690 046	59 390 225	61 279 209	214 012 623	0.00
Cost per woman (excluding NICU and SCN)							
Diet and/or PA	0	3	6	9	12	NA	NA
Diet	226	234	241	249	257	NA	NA
PA	−108	−107	−107	−106	−105	NA	NA
Diet and PA	89	93	98	103	108	NA	NA
Unstructured	227	234	242	250	258	NA	NA

Abbreviations: A$, Australian dollar; NICU, neonatal intensive care unit; NA, not applicable; PA, physical activity; ROI, return on investment; SCN, special care nursery.

^a^ Values can be converted from Australian dollars to US dollars by multiplying by exchange rate (0.739 as of March 22, 2022).^15^

^b^ ROI ratio = total cost savings from intervention/cost of intervention. Prices are as for the year in which they are incurred, no discounting is applied.

Segregating health costs for outpatient (MBS, PBS funded) and inpatient admission (hospital funded) costs showed that 96% of costs savings will be attributable to hospitals. Even with the exclusion of NICU and SCN costs, hospitals will receive 82% of cost savings.

### Sensitivity Analyses

#### Scenario Analysis

Exclusion of NICU and SCN costs from the base case analysis removed a substantial cost offset, with implementation of the lifestyle intervention no longer found to be cost saving. The total cost per woman for the diet and/or physical activity intervention was estimated to be A$12 ($9) in 2026, with a total budget impact of A$67 million ($50 million) for the 5-year nationwide intervention rollout (ROI: 0.97) (Table 3).

Using the Maternity1000 Database, scenario 1 produced an ROI of 5.20 and scenario 2’s ROI was 4.11 (eTable 12 in the Supplement). When NICU and SCN costs were excluded, the ROI ratios dropped to 1.01 (scenario 1) and 1.10 (scenario 2). These ROI ratios were very similar to those returned by the model in the base case, both with and without NICU and SCN, suggesting that the external validity of the model to alternative Australian data sources is good.

#### Deterministic Sensitivity Analysis

**Figure 1.**
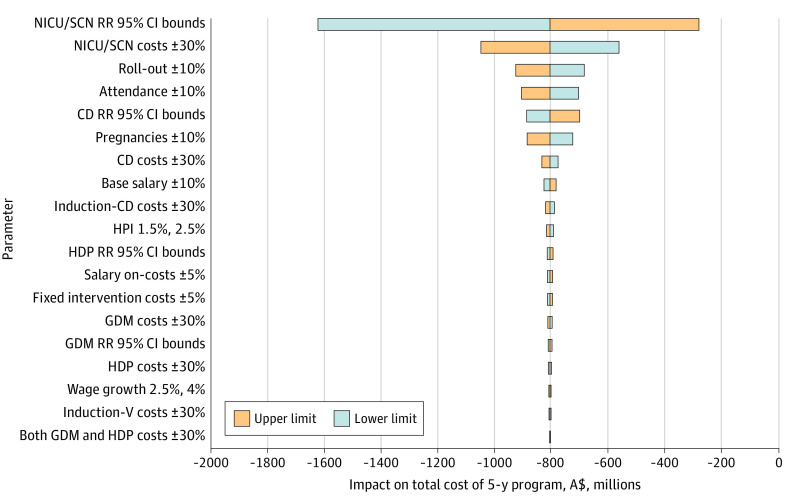
Tornado Chart Summarizing Deterministic Sensitivity Analysis Abbreviations: CD, cesarean delivery; GDM, gestational diabetes; HDP, hypertensive disease in pregnancy; HPI, health price index; Induction-CD, induction of labor with cesarean; Induction-V, induction of labor with vaginal birth; NICU/SCN, neonatal intensive care unit and/or special care nursery; RR, risk ratio.

The results of the 1-way sensitivity analysis are presented in a tornado diagram in Figure 1. The most influential parameter was the risk ratio for NICU and/or SCN admission, followed by NICU and/or SCN weighted mean cost (plus or minus 30%). Importantly, in the base case, aggregate estimated budget impact remained cost saving for all parameter ranges examined.

**Figure 2.**
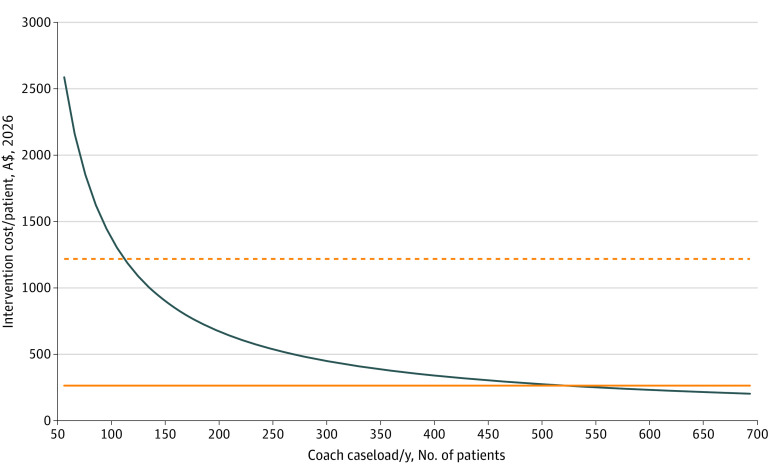
Two-Way Sensitivity Analysis Showing Cost of Intervention per Woman per Year According to Total Caseload per Coach, and Mean Cost Savings From Intervention per Woman per Year Horizontal lines indicate mean cost savings from intervention per woman per year: dashed yellow line includes neonatal ICU and/or special care nursery; solid yellow line excludes neonatal ICU and/or special care nursery.

Two-way sensitivity analysis found that a minimum caseload of 108 women per coach per year was required for the intervention to be cost neutral. If NICU and SCN outcomes and costs are excluded, a minimum of 525 women per coach per year is required (Figure 2). Using the base case assumption of 500 women per coach per year, 157 health coaches will be required to staff the program Australia-wide in the first year of the rollout, increasing to 475 in 2025.

#### Probabilistic Sensitivity Analysis

Probabilistic sensitivity analysis was conducted for the base case. The probability of the program being cost saving when NICU and SCN costs were included was 93.3%, with a 95% CI of −A$129 million (−$95 million) to A$1639 million ($1211 million). When NICU and SCN costs were excluded, the probability of cost saving was 47.5% (95% CI, −A$134 million [−$99 million] to A$150 million [$111 million]).

## Discussion

This economic evaluation’s BIA estimates the cost of the integration of lifestyle interventions into routine antenatal care, with likely cost savings and strong return on investment for health funders. Costs of intervention delivery were projected to be completely offset by savings likely to be achieved through reduced frequency of adverse pregnancy outcomes, providing a 5-fold return on investment in intervention scale-up. This builds on other recent research that found that antenatal lifestyle interventions are both cost-effective and associated with improved clinical outcomes.^4,9,12^ Cost savings estimated using our Australian example could be replicated in US and other international settings, and these can be explored using the open source decision model.

There is international concern around increasing rates of iatrogenic birth in high-income countries,^23^ the associated potential for long-term negative health impacts,^24^ and the costs of providing maternity care.^25^ Transforming the provision of maternity care to focus on providing preventive health care programs to address maternal obesity, gestational diabetes, and HDP—which are key but modifiable risk factors for iatrogenic birth and associated high health care costs—will be essential for improving value.^25,26^

Together with systematic review and cost-effectiveness findings,^4,9,12^ the results of this BIA strongly support integrating lifestyle interventions into routine antenatal care for all pregnant women. While beyond the scope of this study, higher incidence of maternal obesity, gestational diabetes, and HDP in particular subgroups (indigenous women, socioeconomic disadvantage) may support prioritizing intervention rollout in health services serving these population demographics.^6,27^ If program budgets are constrained in the longer term, it may be reasonable to limit programs to nulliparous women, ensuring equitable access to first-time mothers ahead of repeat programs for women in subsequent pregnancies. Cost outcomes from such policy decisions would require further modeling. Future research is funded and under way to use robust predictive models to identify high risk women most likely to benefit from lifestyle intervention.

Successful implementation and scale-up of the intervention could be affected by multiple barriers. Staffing a routine antenatal lifestyle intervention in Australia (with a total population of approximately26 million) will require employment of around 500 health coaches nationwide. Attraction and retention of this substantial skilled workforce may be subject to structural labor market barriers, although flexibility in the professional qualifications and level of experience required should mitigate this issue to some extent. While substantial, the total staff requirement for the program represents less than 1% of the total dietician, midwife, and physiotherapist workforce in Australia.^28,29^ Access to appropriate training is essential to ensure that health coaches have the skills and experience required to confidently discuss gestational weight gain and other sensitive topics with pregnant women.^21^ This may include building on train-the-trainer models that have upscaled lifestyle programs in diabetes prevention.^30^ As scale-up proceeds, it is recommended that ongoing fidelity and effectiveness data are collected to determine whether outcomes in routine clinical practice are similar to those observed in clinical trials.

The strengths of this study include the multiple scenarios examined and data sources used, enabling us to be confident in the external validity of the findings. In particular, examination of individual patient cost data captures the distribution of health costs in the target population. The flexible approach to intervention design and costing is another key strength, allowing health services to tailor intervention delivery to their population provided key elements essential to intervention efficacy are incorporated. Importantly, our model has included training and implementation costs that are often overlooked in cost-effectiveness studies.^31^

### Limitations

This study had some limitations. Although compelling, results of this BIA are also likely to be conservative. The health funder perspective overlooks out-of-pocket expenses for pregnant women, including lost productivity and income. To reduce uncertainty, we have included only health cost offsets that can be confidently allocated a price. The inclusion of NICU and SCN costs in the model substantially affected return on investment; however, inclusion is justified given that this is a health service incurred cost that is a component of birth care (direct outcome of birth event) and part of the intrapartum period.^8^ To provide transparency, modeling results both incorporating and excluding NICU and SCN costs have been provided. The base case model also assumes that probability of NICU and/or SCN admission was the same for all maternal health states as individual patient level data was not available for this outcome. This assumption is conservative because cesarean birth is more likely to result in NICU and/or SCN admission, and cesarean birth is more common for women diagnosed with gestational diabetes and HDP. The reduction in SCN and/or NICU admissions achieved through intervention is therefore likely underestimated in the model. There may be minor variability in cost savings to alternate funding pools (less than 5% of total) for public hospitals that absorb the cost of outpatient specialist appointments, rather than billing the MBS for these services.

## Conclusions

This economic evaluation’s findings suggest that implementation of routine structured antenatal lifestyle interventions Australia-wide to improve healthy lifestyle and limit excess gestational weight gain is likely to provide strong return on investment for health funders. This, together with the projected health benefits, provides a compelling argument that this intervention should be made available to all Australian women during pregnancy.

## Notes

Supplement. **eFigure 1.** Decision Tree Model **eTable 1.** Patient Pathway Costs for Gestational Diabetes Mellitus (GDM) **eTable 2.** Patient Pathway Costs for Hypertensive Disease In Pregnancy (HDP) **eTable 3.** Patient Pathway Costs for Gestational Diabetes And Hypertensive Disease in Pregnancy **eTable 4.** Unit Costs for Consultations, Hospital Admissions, Pathology, Sonography and Medication **eTable 5.** Birth Costs According to Mode of Delivery **eTable 6.** Neonatal Intensive Care and Special Care Nursery DRGs (2018-19) **eTable 7.** Weighted Mean Costs by Mode of Delivery From the Maternity1000 Dataset (AUD) **eTable 8.** Antenatal and NICU/SCN Mean Costs From Maternity1000 Dataset (AUD, 2022 Prices) **eTable 9.** Published Enterprise Bargaining Agreement Salaries for Health Professionals Delivering Intervention (2020-21) **eTable 10.** Intervention Effect Risk Ratios for All Intervention Groupings **eTable 11.** Proportion of Birth Outcomes by Baseline Health State for Pregnancies From the Maternity1000 Dataset **eTable 12.** Budget Impact Analysis Using Input Parameters From the Maternity1000 Database
eReferences
