# Supplementary material for: Projected Return on Investment From Implementation of a Lifestyle Intervention to Reduce Adverse Pregnancy Outcomes
Source: JAMA Netw Open. 2022 Sep 6;5(9):e2230683. doi: 10.1001/jamanetworkopen.2022.30683 (PMC9449797; doi:10.1001/jamanetworkopen.2022.30683)
Supplement: Supplement. — eFigure 1. Decision Tree Model eTable 1. Patient Pathway Costs for Gestational Diabetes Mellitus (GDM) eTable 2. Patient Pathway Costs for Hypertensive Disease In Pregnancy (HDP) eTable 3. Patient Pathway Costs for Gestational Diabetes And Hypertensive Disease in Pregnancy eTable 4. Unit Costs for Consultations, Hospital Admissions, Pathology, Sonography and Medication eTable 5. Birth Costs According to Mode of Delivery eTable 6. Neonatal Intensive Care and Special Care Nursery DRGs (2018-19) eTable 7. Weighted Mean Costs by Mode of Delivery From the Maternity1000 Dataset (AUD) eTable 8. Antenatal and NICU/SCN Mean Costs From Maternity1000 Dataset (AUD, 2022 Prices) eTable 9. Published Enterprise Bargaining Agreement Salaries for Health Professionals Delivering Intervention (2020-21) eTable 10. Intervention Effect Risk Ratios for All Intervention Groupings eTable 11. Proportion of Birth Outcomes by Baseline Health State for Pregnancies From the Maternity1000 Dataset eTable 12. Budget Impact Analysis Using Input Parameters From the Maternity1000 Database eReferences [file jamanetwopen-e2230683-s001.pdf]

Supplemental Online Content

Lloyd M, Teede H, Bailey C, Callander E, Ademi Z. Projected return on investment from implementation of a lifestyle intervention to reduce adverse pregnancy outcomes. *JAMA Netw Open.* 2022;5(9):e2230683. doi:10.1001/jamanetworkopen.2022.30683

eFigure 1. Decision Tree Model

eTable 1. Patient Pathway Costs for Gestational Diabetes Mellitus (GDM)

eTable 2. Patient Pathway Costs for Hypertensive Disease In Pregnancy (HDP)

eTable 3. Patient Pathway Costs for Gestational Diabetes And Hypertensive Disease in Pregnancy

eTable 4. Unit Costs for Consultations, Hospital Admissions, Pathology, Sonography and Medication

eTable 5. Birth Costs According to Mode of Delivery

eTable 6. Neonatal Intensive Care and Special Care Nursery DRGs (2018-19)

eTable 7. Weighted Mean Costs by Mode of Delivery From the Maternity1000 Dataset (AUD)

eTable 8. Antenatal and NICU/SCN Mean Costs From Maternity1000 dataset (AUD, 2022 prices)

eTable 9. Published Enterprise Bargaining Agreement Salaries for Health Professionals Delivering Intervention (2020-21)

eTable 10. Intervention Effect Risk Ratios for All Intervention Groupings

eTable 11. Proportion of Birth Outcomes by Baseline Health State for Pregnancies From the Maternity1000 Dataset

eTable 12. Budget Impact Analysis Using Input Parameters From the Maternity1000 Database

eReferences

This supplemental material has been provided by the authors to give readers additional information about their work.

© 2022 Lloyd M et al. *JAMA Network Open.*

eFigure 1. Decision tree model

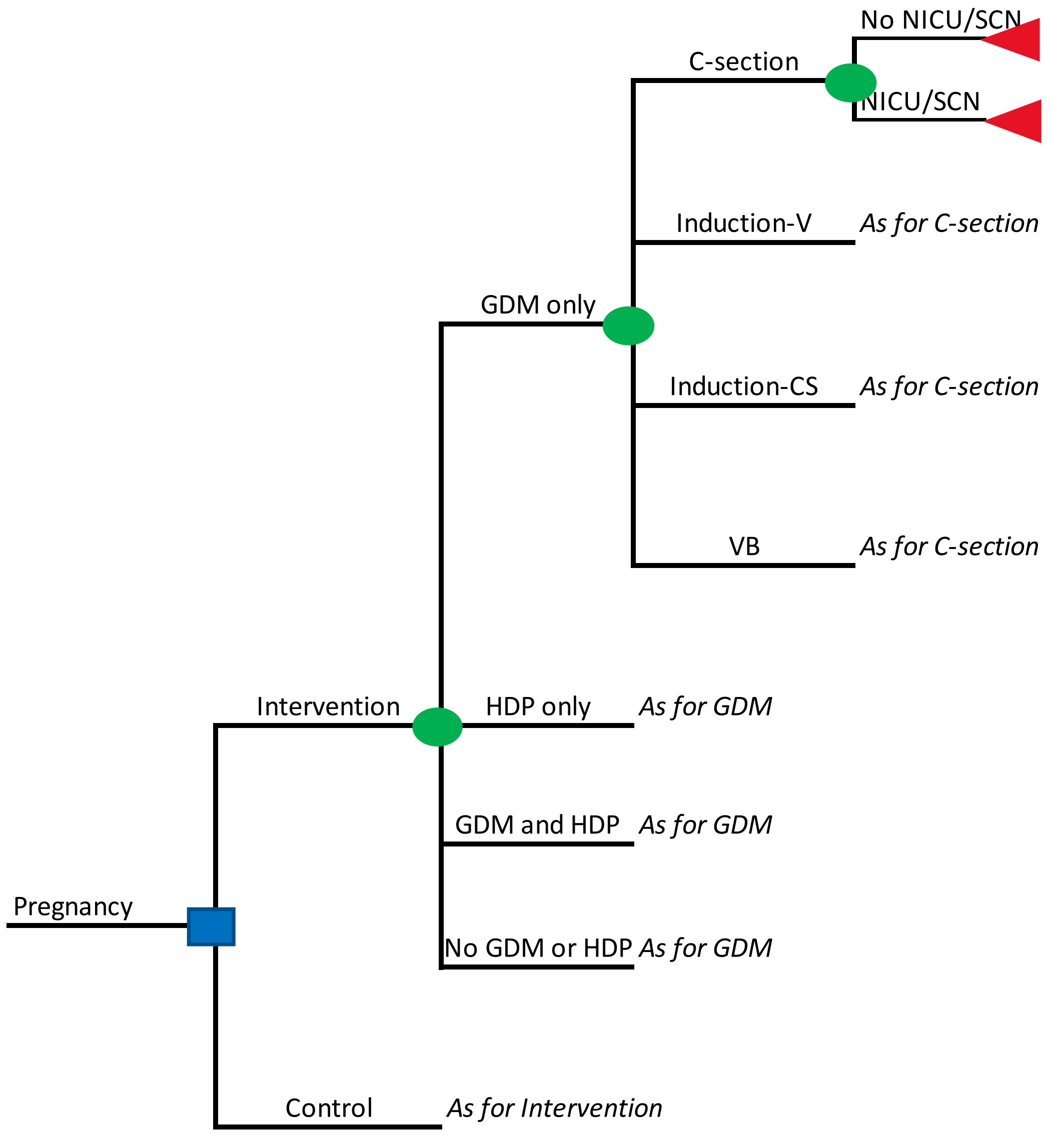

Abbreviations: Abbreviations: C-section: caesarean section (planned or spontaneous onset labour); GDM: gestational diabetes; HDP: Hypertensive disease in pregnancy; Induction-CS: induction of labour with caesarean section, Induction-V: induction of labour with vaginal birth; NICU/SCN: neonatal intensive care unit / special care nursery; VB: spontaneous vaginal birth.

© 2022 Lloyd M et al. *JAMA Network Open.*

Pathways for antenatal management of Gestational Diabetes and Hypertension During Pregnancy

eTable 1: Patient pathway costs for gestational diabetes mellitus (GDM)

	Units per subject	Total cost per subject (AUD)	
		**2019 prices**	**2022 prices**
**Consultations**			
Endocrinologist (initial)	1	153.15	161.63
Endocrinologist (subsequent)	2.72	208.50	220.05
Obstetrician (visits in excess of standard antenatal care)	2.5	82.38	86.94
Dietician (individual sessions)	0.5	31.13	32.85
Dietician (group sessions)	1	19.90	21.00
Diabetes educator (individual sessions)	1	62.25	65.70
Diabetes educator (group sessions)	1	19.90	21.00
**Hospital admission**			
Antenatal hospital admission, excess per person with GDM compared to no GDM[1]		27.00	28.50
**Pathology**			
Glucose monitoring kit including four month supply of lancets and test strips (for use by patient)	1	156.96	165.65
**Sonography**			
Ultrasound to assess foetal health at approximately 32-34 weeks gestation	1	115.00	121.37
Ultrasound to assist with the decision regarding timing and mode of birthing	0.5	57.50	60.68
Antenatal cardiotocography in the management of high risk pregnancy	0.5	18.33	19.35
**Medication**			
Insulin (twice daily from 28-40 weeks gestation)	0.31	102.59	108.27
**Total**		**1054.59**	**1112.99**

Adapted from Bailey et al. 2020, Supplementary Tables.[2]

© 2022 Lloyd M et al. *JAMA Network Open.*

eTable 2: Patient pathway costs for hypertensive disease in pregnancy (HDP)

	Units	Cost per subject (AUD)	
		2019 prices	2022 prices
**Consultations**			
Specialist- first appointment	1	153.15	161.63
Subsequent appointment	1	76.65	80.89
Midwife – outpatients, doctors review tests	4	78.40	82.74
**Hospital admission**			
Antepartum admission	1	834.00	880.19
**Medication**			
Medication for blood pressure- estimate for 3 months	1	64.08	67.63
**Sonography**			
Ultrasound	2	230.00	242.74
CTG	2	73.30	77.36
**Pathology**			
Blood tests	4	343.20	362.21
Urine test	2	$37.90	40.00
**Total**		**1922.98**	**2029.47**

Note: for hypertensive disease in pregnancy, some health service users will require little or no medication/services, whilst some will require extensive services. This table has attempted to estimate a mean number of units across all health service users, and was validated by comparing to a previous publication[3].

Adapted from Bailey et al. 2020, Supplementary Tables. [2]

© 2022 Lloyd M et al. *JAMA Network Open.*

eTable 3: Patient pathway costs for gestational diabetes and hypertensive disease in pregnancy

	Units per subject	Total cost per subject (AUD)	
		2019 prices	2022 prices
**Consultations**			
Specialist	2	306.30	323.26
Specialist (subsequent)	2.72	208.50	220.05
Obstetrician (visits in excess of standard antenatal care)	2.5	82.38	86.94
Dietician (individual sessions)	1	62.25	65.70
Dietician (group sessions)	1	19.90	21.00
Diabetes educator (individual sessions)	2	124.50	131.39
Diabetes educator (group sessions)	1	19.90	21.00
**Hospital admission**			
Antepartum admission	1	834.00	880.19
**Pathology**			
Glucose monitoring kit including four month supply of lancets and test strips (for use by patient)	1	156.96	165.65
Oral Glucose Tolerance Test (6-8 weeks post-partum)	1	18.95	20.00
Urine test	2	19.40	20.47
Blood tests	4	343.20	362.21
**Sonography**			
Ultrasound to assess fetal health at approximately 32-34 weeks gestation	1	115.00	121.37
Ultrasound to assist with the decision regarding timing and mode of birthing	2	230.00	242.74
Antenatal cardiotocography in the management of high risk pregnancy	2	73.30	77.36
**Medication**			
Insulin (twice daily from 28-40 weeks gestation)	0.31	102.59	108.27
Medication for blood pressure- estimate for 3 months	1	64.08	67.63
**Total**		**2781.21**	**2935.23**
Adapted from Bailey et al. 2020, Supplementary Tables.[2]			

© 2022 Lloyd M et al. *JAMA Network Open.*

eTable 4: Unit costs for consultations, hospital admissions, pathology, sonography and medication

Service	Source	Cost (AUD, 2019 prices)
**Consultations**		
Specialist- initial consultation	MBS [4] Item 110	153.15
Specialist- subsequent consultation	MBS Item 116	76.65
Obstetrician (visits in excess of standard antenatal care)	MBS Item 16404	32.95
Dietician (individual sessions)	MBS Item 10954	62.25
Dietician (group sessions)	MBS Item 81105	19.90
Diabetes educator (individual sessions)	MBS Item 10951	62.25
Diabetes educator (group sessions)	MBS Item 81105	19.60
Extra pre-partum visit with midwife	MBS Item 82105	32.30
Hospital admission		
**Antepartum admission**	Estimated number of days and costs per day as per advice from clinicians in Australia, and UK data.[1] 1.33 days on average per person at GBP379.22[a]	834.00
**Pathology**		
Glucose monitoring kit including four month supply of lancets and test strips (for use by patient)	Monitor = $41.49, plus 4 times test strips (100 per packed, assuming testing three times a day, therefore on packet per month) $47.49 each	156.96
Oral Glucose Tolerance Test	MBS: 66542	18.95
Urine test for protein	MBS: 66500	9.70
Blood tests		
Full blood examination	MBS: 65070	16.95
EUC - electrolytes, urea, creatine	MBS: 66509	15.65
Liver function tests	MBS: 66512	17.70
Coagulation studies	MBS: 65129	35.50
**Sonography (pre-natal)**		
Ultrasound to assess foetal health at approximately 32-34 weeks gestation	MBS Item 55721	115.00
Antenatal cardiotocography in the management of high risk pregnancy,	MBS Item 16514	36.65
**Medication**		
Insulin (twice daily from 28-40 weeks gestation)	PBS [5] 1761Q- Insulin Isophane, Injections (human) 100 units per mL, 3 mL, 5, (approx. 3 months supply)	120.90
Medication for blood pressure	PBS Item 1629R- Methyldopa or similar (Labetalol, Nifedipine, or Hydralazine), for three months, estimate, three month supply at 21.36 per month	64.08

[a]This figure was then adjusted for resource utilisation and price of healthcare purchases using purchasing power parity from GBP to AUD.

Abbreviations: MBS: Medicare Medical Benefits Schedule, PBS: Medicare Pharmaceutical Benefits Schedule

Adapted from Bailey et al. 2020, Supplementary Tables. [2]

© 2022 Lloyd M et al. *JAMA Network Open.*

Delivery costs

eTable 5: Birth costs according to mode of delivery

Delivery mode	DRG	Description	Separations (n)	Item cost[a]	Aggregate cost (AUD)	Weighted cost (AUD)	
						2019	2022
Vaginal birth	O02A	Vaginal Delivery W GIs, Major Complexity	1,995	14,250	28,429,426		
	O02B	Vaginal Delivery W GIs, Minor Complexity	4,623	9,901	45,771,109		
	O60A	Vaginal Delivery, Major Complexity	25,317	9,999	253,143,900		
	O60B	Vaginal Delivery, Intermediate Complexity	64,883	6,780	439,919,566		
	O60C	Vaginal Delivery, Minor Complexity	53,406	4,986	266,279,534		
	**Total**		**150,224**		**1,033,543,536**	**6880**	**7261**
C-section	O01A	Caesarean Delivery, Major Complexity	10,281	18,341	188,561,356		
	O01B	Caesarean Delivery, Intermediate Complexity	30,939	12,900	399,105,629		
	O01C	Caesarean Delivery, Minor Complexity	30,142	10,476	315,755,292		
	**Total**		**71,362**		**903,422,277**	**12660**	**13361**

[a]Diagnosis-related group (DRG) data from 2018/19 were used for delivery costs, and adjusted to 2022 prices using the health price index[6].

Induction costs were estimated to be a proportional increase from the cost of an uncomplicated vaginal birth, and half of induced births were estimated to also have an epidural.[7] Inductions with and without an epidural were estimated as 1.54 and 1.21 times that of an uncomplicated vaginal birth. Using this methodology, induction with vaginal birth was estimated as AUD9460, and induction costs separately were estimated at AUD2580 (2019 prices).

© 2022 Lloyd M et al. *JAMA Network Open.*

eTable 6: Neonatal Intensive Care and Special Care Nursery DRGs (2018-19)

DRG	DRG Description	Separations (n)	Item cost[a]	Aggregate item cost
P01Z	Neonate W Sig GI/Vent>=96hrs, Died or Transfer to Acute Facility <5Days	249	14,368	3,577,544
P02Z	Cardiothoracic and Vascular Interventions for Neonates	229	172,102	39,411,460
P03A	Neonate, AdmWt 1000-1499g W Significant GI/Vent>=96hrs, Major Complexity	412	157,996	65,094,201
P03B	Neonate, AdmWt 1000-1499g W Significant GI/Vent>=96hrs, Minor Complexity	551	90,726	49,989,772
P04A	Neonate, AdmWt 1500-1999g W Significant GI/Vent>=96hrs, Major Complexity	138	137,583	18,986,392
P04B	Neonate, AdmWt 1500-1999g W Significant GI/Vent>=96hrs, Minor Complexity	459	61,190	28,086,296
P05A	Neonate, AdmWt 2000-2499g W Significant GI/Vent>=96hrs, Major Complexity	119	135,245	16,094,149
P05B	Neonate, AdmWt 2000-2499g W Significant GI/Vent>=96hrs, Minor Complexity	277	46,113	12,773,330
P06A	Neonate, AdmWt >=2500g W Significant GI/Vent>=96hrs, Major Complexity	543	108,324	58,820,181
P06B	Neonate, AdmWt >=2500g W Significant GI/Vent>=96hrs, Minor Complexity	777	34,366	26,702,666
P07Z	Neonate, AdmWt <750g W Significant GIs	57	353,545	20,152,044
P08Z	Neonate, AdmWt 750-999g W Significant GIs	42	248,346	10,430,536
P60A	Neonate W/O Sig GI/Vent>=96hrs, Died/Transfer Acute Facility <5 Days, Maj Comp	838	7,523	6,304,148
P60B	Neonate W/O Sig GI/Vent>=96hrs, Died/Transfer Acute Facility <5 Days, Min Comp	3,980	4,355	17,331,404
P61Z	Neonate, AdmWt <750g W/O Significant GI procedure	312	228,916	71,421,649
P62A	Neonate, AdmWt 750-999g W/O Significant GIs, Major Complexity	282	215,016	60,634,555
P62B	Neonate, AdmWt 750-999g W/O Significant GIs, Minor Complexity	232	128,638	29,844,058
P63A	Neonate, AdmWt 1000-1249g W/O Significant GI/Vent>=96hrs, Major Complexity	109	61,285	6,680,068
P63B	Neonate, AdmWt 1000-1249g W/O Significant GI/Vent>=96hrs, Minor Complexity	128	14,764	1,889,798
P64A	Neonate, AdmWt 1250-1499g W/O Significant GI/Vent>=96hrs, Major Complexity	341	54,552	18,602,333
P64B	Neonate, AdmWt 1250-1499g W/O Significant GI/Vent>=96hrs, Minor Complexity	277	37,351	10,346,286
P65A	Neonate, AdmWt 1500-1999g W/O Significant GI/Vent>=96hrs, Extreme Comp	571	54,166	30,928,660
P65B	Neonate, AdmWt 1500-1999g W/O Significant GI/Vent>=96hrs, Major Complexity	498	42,592	21,210,712
P65C	Neonate, AdmWt 1500-1999g W/O Significant GI/Vent>=96hrs, Intermediate Comp	1,351	32,057	43,309,429
P65D	Neonate, AdmWt 1500-1999g W/O Significant GI/Vent>=96hrs, Minor Complexity	1,491	25,211	37,589,794
P66A	Neonate, AdmWt 2000-2499g W/O Significant GI/Vent>=96hrs, Extreme Comp	1,619	31,472	50,953,599
P66B	Neonate, AdmWt 2000-2499g W/O Significant GI/Vent>=96hrs, Major Complexity	3,069	18,925	58,081,810
P66C	Neonate, AdmWt 2000-2499g W/O Significant GI/Vent>=96hrs, Intermediate Comp	1,995	12,667	25,270,453
P66D	Neonate, AdmWt 2000-2499g W/O Significant GI/Vent>=96hrs, Minor Complexity	2,642	7,158	18,910,340
P67A	Neonate, AdmWt >=2500g W/O Sig GI/Vent>=96hrs, <37 Comp Wks Gest, Extr Comp	1,132	28,042	31,743,880
				Cont.

© 2022 Lloyd M et al. *JAMA Network Open.*

DRG	DRG Description	Separations (n)	Item cost	Aggregate item cost
P67B	Neonate, AdmWt >=2500g W/O Sig GI/Vent>=96hrs, <37 Comp Wks Gest, Maj Comp	786	17,109	13,447,782
P67C	Neonate, AdmWt >=2500g W/O Sig GI/Vent>=96hrs, <37 Comp Wks Gest, Int Comp	1,175	13,701	16,098,777
P67D	Neonate, AdmWt >=2500g W/O Sig GI/Vent>=96hrs, <37 Comp Wks Gest, Min Comp	2,925	8,800	25,739,885
P68A	Neonate, AdmWt >=2500g W/O Sig GI/Vent>=96hrs, >=37 Comp Wks Gest, Ext Comp	4,473	16,198	72,451,889
P68B	Neonate, AdmWt >=2500g W/O Sig GI/Vent>=96hrs, >=37 Comp Wks Gest, Maj Comp	8,134	7,613	61,921,575
P68C	Neonate, AdmWt >=2500g W/O Sig GI/Vent>=96hrs, >=37 Comp Wks Gest, Int Comp	11,044	5,387	59,492,961
P68D	Neonate, AdmWt >=2500g W/O Sig GI/Vent>=96hrs, >=37 Comp Wks Gest, Min Comp	19,875	3,746	74,442,444
**Total**		**53,257**		**1,214,766,860**
**Weighted cost**			**2019 price**	**22,810**
			**2022 price**	**24,073**

[a]Diagnosis-related group (DRG) data from 2018/19 were used for delivery costs, and adjusted to 2022 prices using the health price index[6].

© 2022 Lloyd M et al. *JAMA Network Open.*

eTable 7. Weighted mean costs by mode of delivery from the Maternity1000 dataset* (AUD)

Delivery costs	GDM	HDP	Both	Neither	Weighted mean cost	
	2019 prices				2019 prices	2022 prices
Vaginal delivery	8,778	9,496	9,656	8,610	9,537	9,883
Caesarean section cost premium[a]	6,333	6,567	8,005	5,857	5,980	6,197
Induction-vaginal birth cost premium[a]	1,100	1,100	1,100	1,100	1,100	1,140
Induction-caesarean section cost premium[a]	7,433	7,667	9,105	6,957	7,080	7,337

[a]Cost premium is the additional cost of the delivery mode over and above the cost the of a spontaneous vaginal delivery.

Abbreviations: GDM: gestational diabetes mellitus; HDP: hypertensive disease in pregnancy.

eTable 8. Antenatal and NICU/SCN mean costs from Maternity1000 dataset* (AUD, 2022 prices)

	Mean cost
GDM antenatal costs	2,532
HDP antenatal costs	2,399
GDM and HDP	6,474
NICU/SCN	17,259

Abbreviations: GDM: gestational diabetes mellitus; HDP: hypertensive disease in pregnancy; NICU/SCN: neonatal intensive care unit/ special care nursery.

*Maternity1000 utilises the Queensland Perinatal Data Collection and Queensland Birth Registry to identify mothers who gave birth in Queensland, Australia, between July 2012 and June 2019 (n~360,000). Records of all included individuals were linked to the Queensland Hospital Admitted Patient Data Collection, Non-admitted Patient Data Collection, Deaths Registry, Emergency Department Information System and Hospital and Health Service Funding and Costing Unit records between 1 July 2012 and 30 June 2019. Records were linked by the Australian Institute of Health and Welfare to their corresponding MBS and PBS claims records. All costs in the dataset have been inflated to 2019-20 prices.

© 2022 Lloyd M et al. *JAMA Network Open.*

Intervention Costs

eTable 9: Published Enterprise Bargaining Agreement salaries for health professionals delivering intervention (AUD 2020-21).

	**Victoria**		**Queensland**		**NSW**		**Tasmania**	
	Grade	Salary	Grade	Salary	Grade	Salary	Grade	Salary
**Dietician**	Level 2, Year 2	89,908	HP3.3	87,993	Level 2, Year 2	86,274	Level 2, Year 2	82,492
**Physiotherapist**	Level 2, Year 2	87,204	HP3.3	87,993	Level 2, Year 2	86,274	Level 2, Year 2	82,492
**Midwife**	RN, Year 4	74,672	Band 5, Step 4	84,761	RN, Year 4	76,388	RN Grade 3, Year 4	75,207
**Selected midpoint full-time equivalent salary (AUD; 2021 prices)**								**80,000**

Note: A mid-point salary was selected for each profession (generally the mid-point of the second salary band level – representing staff with on average 4-6 years professional experience).

© 2022 Lloyd M et al. *JAMA Network Open.*

Intervention Effects

eTable 10. Intervention effect risk ratios for all intervention groupings

	**Risk ratio**
Diet+/-PA intervention:	
GDM risk ratio	0.670
HDP risk ratio	0.737
GDM and HDP risk ratio	0.670
C-section risk ratio	0.929
Induction risk ratio	1
NICU/SCN risk ratio	0.795
Diet only intervention:	
GDM risk ratio	0.656
HDP risk ratio	0.812
GDM and HDP risk ratio	0.656
C-section risk ratio	1.049
Induction risk ratio	1
NICU risk ratio	0.700
Diet+PA intervention:	
GDM risk ratio	0.749
HDP risk ratio	0.764
GDM and HDP risk ratio	0.749
C-section risk ratio	0.968
Induction risk ratio	1
NICU risk ratio	0.956
PA only intervention:	
GDM risk ratio	0.624
HDP risk ratio	0.675
GDM and HDP risk ratio	0.624
C-section risk ratio	0.879
Induction risk ratio	1
NICU risk ratio	0.730
Unstructured intervention:	
GDM risk ratio	1.025
HDP risk ratio	1.124
GDM and HDP risk ratio	1.025
C-section risk ratio	0.986
Induction risk ratio	1
NICU risk ratio	1.092

Odds ratios taken from Teede et al. 2021[8] and converted to Risk Ratios as per the method outlined in Bailey et al. 2020.[2]

Abbreviations: GDM: gestational diabetes mellitus; HDP: hypertensive disease in pregnancy; NICU: neonatal intensive care unit; PA: physical activity.

© 2022 Lloyd M et al. *JAMA Network Open.*

eTable 11. Proportion of birth outcomes by baseline health state for pregnancies from the Maternity1000 dataset

	C-section	Induction-v	Induction-c	VB	SCN	NICU	Total SCN/NICU
GDM	0.3287	0.3490	0.0758	0.2466	0.2579	0.0227	0.2806
HDP	0.2590	0.4726	0.1378	0.1306	0.2292	0.0222	0.2514
Both	0.4248	0.3224	0.1307	0.1220	0.3813	0.0240	0.4053
None	0.2918	0.1962	0.0436	0.4681	0.1508	0.0182	0.1690
All groups	0.2946	0.2189	0.0495	0.4367	0.1637	0.0188	0.1824

Abbreviations – C-section: caesarean section (planned or spontaneous onset labour); GDM: gestational diabetes mellitus; HDP: Hypertension during pregnancy; Induction-c: Induction of labour with c-section; Induction-v: Induction of labour with vaginal birth; NICU: Neonatal intensive care unit; SCN: Special care nursery; VB: spontaneous vaginal birth.

© 2022 Lloyd M et al. *JAMA Network Open.*

eTable 12: Budget Impact Analysis using input parameters from the Maternity1000 Database

		2022	2023	2024	2025	2026	TOTAL	ROI ratio
	Including NICU/SCN							
**Scenario 1[a]**	**Total cost**	-78,171,806	-133,160,266	-190,155,372	-248,910,233	-252,960,118	-903,357,794	5.20
	**Cost per woman**	-995	-1,012	-1,030	-1,047	-1,065		
	Excluding NICU/SCN							
	**Total cost**	-813,380	-1,014,638	-909,736	-472,275	263,563	-2,946,465	1.01
	**Cost per woman**	-10	-8	-5	-2	1		
	Including NICU/SCN							
**Scenario 2[b]**	**Total cost**	-57,946,563	-98,610,993	-140,677,389	-183,956,507	-186,755,172	-667,946,624	4.11
	**Cost per woman**	-738	-750	-762	-774	-786		
	Excluding NICU/SCN							
	**Total cost**	-2,484,999	-3,870,140	-4,999,099	-5,840,710	-5,208,286	-22,403,234	1.10
	**Cost per woman**	-32	-29	-27	-25	-22		

[a]Scenario 1, model parameters were updated for health states and birth outcomes under usual care with baseline risks obtained from Maternity1000 (eTable 11).

[b]Scenario 2 also included parameter distributions for health costs derived from individual patient level cost data from Maternity1000 (eTable 7-8).

© 2022 Lloyd M et al. *JAMA Network Open.*

eReferences

1. Australian Insititute of Health and Welfare. Diabetes in Pregnancy: Its Impact on Australian Women and Their Babies. Diabetes Series No. 14. Cat. No. CVD 52 2010 [Available from: https://www.aihw.gov.au/reports/diabetes/diabetes-pregnancy-impact-on-women-babies/contents/table-of-contents.
2. Bailey C, Skouteris H, Harrison CL, Boyle J, Bartlett R, Hill B, et al. Cost effectiveness of antenatal lifestyle interventions for preventing gestational diabetes and hypertensive disease in pregnancy. Pharmacoecon Open. 2020;4(3):499-510.
3. Fox H, Callander EJ. The cost of Hypertensive Disorders of Pregnancy to the Australian healthcare system. Pregnancy Hypertens. 2020;21:197-9.
4. Australian Department of Health. Medicare Benefits Schedule. MBS Online. 2018 [Available from: http://www.mbsonline.gov.au/internet/mbsonline/publishing.nsf/Content/Downloads-201811.
5. Australian Department of Health. Schedule of Pharmaceutical Benefits. Australian Government. Canberra; 2019.
6. Australian Institute of Health and Welfare. Health Expenditure Australia 2017-18. Australian Government. Canberra; 2019.
7. Tracy SK, Tracy MB. Costing the cascade: estimating the cost of increased obstetric intervention in childbirth using population data. Brit J Obstet Gynecol. 2003;110(8):717-24.
8. Teede HJ, Bailey C, Moran LJ, Khomami MB, Enticott J, Ranasinha S, et al. Association of antenatal diet and physical activity–based interventions with gestational weight gain and pregnancy outcomes: A systematic review and meta-analysis. JAMA Intern Med. 2022;182(2):106-114.

© 2022 Lloyd M et al. *JAMA Network Open.*
